# Quantum random number generators with entanglement for public randomness testing

**DOI:** 10.1038/s41598-019-56706-2

**Published:** 2020-01-13

**Authors:** Janusz E. Jacak, Witold A. Jacak, Wojciech A. Donderowicz, Lucjan Jacak

**Affiliations:** 1 Department of Quantum Technologies, Faculty of Fundamental Problems of Technology, Wrocław University of Science and Technology, Wyb. Wyspiańskiego 27, 50-370 Wrocław, Poland; 2 CompSecur, Pilsudskiego 74/309b, 50-020 Wroclaw, Poland

**Keywords:** Qubits, Quantum information

## Abstract

We discuss a simple idealistic quantum entanglement based protocol for quantum random number generation allowing a trusted third party to publicly perform arbitrarily complex tests of randomness without any violation of the secrecy of the generated bit sequences. The protocol diminishes also an average time of the randomness testing (thus enabling arbitrary shortening of this time with increasing number of entangled qubits).

## Introduction

Quantum random number generators (QRNGs) are aspiring to be a new standard of randomness generators, and not only in cryptography, but also in many other fields like AI, Monte Carlo like simulations, sampling, etc. The pureness of the quantum process, despite its theoretical non-deterministic fundamental unpredictability, still remains, however, the main problem in practical QRNG implementations, including the so-called Device Independent (DI) QRNGs^1–5^.

While some of the protocols extract quantum randomness and discard deterministic components^6,7^ arisen due to quantum processes implementation imperfections, the fidelity of such procedures is not ideal. Just similarly as for the quantum key distribution protocols, which are theoretically unconditionally secure, but in real implementations are always secure only up to some certain level, conditioned by physical implementation shortcomings. Enhancement of the security level usually happens at a cost of lowering of the overall efficiency. Some DI QRNG approaches are limited to specific generating techniques and setups (like e.g., continuous variables approaches)^4,8–10^. The other, like self-testing QRNG protocols, considered to be of the Device Independent approach-type, e.g., in ref. ^11^, allow to separate a deterministic classical component from quantum one resulting in definable confidence levels of generated bit series (e.g. 99% as in^11^). Eventually these issues can be reduced to statistical predictions, like in quantum component within continuous variables approaches or statistical proofs of Bell or Mermin type inequalities violation proving non-classical entanglement and thus quantumness of the protocol^12,13^ (but again statistically and imperfectly, which can be tackled with special protocols for error correction^14,15^, especially such as entanglement purification^14,16–21^). Thus the problem of statistically analyzing the entropy of the source of randomness still remains an important aspect for any physical implementation with inevitable technical imperfections of quantum random generators (e.g.^10,22^).

The measurement of the entropy of the random binary sequence (formally treated as a random variable of all the possible bit string configurations) is not an easy task computationally. Different tools can be considered, including e.g., entropy accumulation theorem^23^ or entropy monitoring^3^. It should be noted that in case of a single bit string there is no access to measuring of the entropy of the source (as this requires many generated bit strings). Instead however if this bit string is long enough, it can be divided into parts that correspond to sampling of the source with shorter bit strings. Therefore measuring entropy of the bit string can be reduced to measuring entropy of the source at the cost of increasing length of the required bit string. As the entropy of the source corresponds to random variable of possible generated bit configurations with frequential probabilities, the solution to problem of measuring this entropy can be implemented by searching all possible patterns occurrences in the bit string. Hence the general problem can be viewed as finding the correlations (or all possible patterns) in the sequence, and on this ground, calculation of the frequential probabilities of given patterns occurrences, which finally allows to calculate the entropy. But, for large random bits sequences computational complexity of classical algorithms for identifying correlations (finding repeated patterns) scales exponentially with the length of the patterns sought for (because the number of such patterns grows exponentially with their lengths). This poses a main difficulty for practical testing of the randomness quality. In an ideal quantum case, the perfect randomness generated should be truly non-deterministic, without any correlations in terms of repeated patterns occurrences, as a consequence of the quantum measurement.

The exponentially growing computational difficulty in the verification of random binary distributions is a well recognized problem and it was the basis of the recent quantum supremacy result presented (2019) by Google and the UC Santa Barbara research team^24^. The Google’s Sycamore superconducting quantum processor with the architecture designed to be applied for this particular problem, introduces a multi-qubit entanglement, in Google’s device – 53-qubit entanglement. This enables a corresponding reduction of the computational complexity at the cost of the entanglement level measured by the number of qubits involved in a multi-qubit entangled state of the processor (this reduction scales exponentially with number of qubits within a multi-qubit entangled state). The architecture of the Google’s quantum processor as shown in Fig. 3 of ref. ^24^ and designed to exponentially diminish the problem of verification of binary sequence random distribution, closely resembles the quantum gate structure of the multi-qubit case of considered in our protocol (earlier patented in 2017^25^ in a conceptual scope as a device with indicated quantum gate structures – cf. Fig. 5.a and .b of the patent), and utilizes the same property as discussed in the present paper. That property enables an arbitrary reduction of the computation time in binary sequence randomness testing at the cost of the increase of the number of multi-entanglement state participating qubits (i.e., of *n*-qubits entanglement state) in the exponential dependence on the entanglement multiplicity (a number of qubits entangled all together). We emphasize that the discussed here advantage of multiple qubits entangled states for randomness testing has been formulated prior to the publication of Sycamore by Google as indicated in the mentioned patent (cf. i.a. paragraphs 19, 77, as well as Figs. 5 and 6^25^).

In principle, the simple entanglement based randomness generation protocols (not involving multi-qubit entanglement, but just limited to two-qubit Bell states) are limited by the barrier of verification of randomness of the resultant binary sequence requiring the exponentially growing computational resources. Testing of the Bell inequalities violation is also a statistical procedure that can be represented as entropy assessments of bit patterns. The particular CHSH inequality test^26^ is present in most entanglement based randomness generation protocols. It verifies whether the CHSH expected value (average value) in measurement is below or above the value of 2 (a classical limit), up to the value of $2\sqrt{2}$, which corresponds to the pure (ideal) Bell state quantum entanglement. This entanglement verification does not, however (in non-ideal case), prove that the generated sequence is truly random in statistical sense (i.e., it does not contain any correlations). In case that the Bell measurement average value is below $2\sqrt{2}$ (any realistic implementation), it is impossible to verify or test randomness of the generated sequence without actually processing the random sequence looking for multiple-bits patterns which is an exponential computational resources consuming task. Utilizing currently known protocols it is also impossible to provide a public proof up to some fidelity level of randomness if the random sequence is to remain secret and not revealed.

The public verification and the proof of the bit sequence being random at a certain level of fidelity can indeed be performed by measuring the entropy of a given generated random sequence, which is however not an easy computational task, as it must concern all the possible patterns, i.e., also including long-range patterns possible to appear in an arbitrarily long sequences. When the generated random bit string size increases (e.g., towards gigabits of random binary stream) then finding its entropy (measured in frequential probability of all possible pattern occurrences in the generated string) is significantly limited by classical computational resources. In those terms, the measurement of the entropy is the same complexity problem as the verification of the randomness, both in fundamental and practical terms and of the same resource consuming character. The proposed in the present paper protocol (even in its non *n*-qubit entanglement generalized version) takes one its advantage form the shift of this cumbersome randomness testing (in principle, equivalent entropy ‘measurement’) to the external public third parties with unlimited resources, in general case. A second, even more crucial its advantage is in the mentioned *n*-qubit entanglement generalization, which allows to exponentially reduce the computational resources needed for binary sequence random distribution verification (recently implemented experimentally by Google). An arrangement of the public validation of the randomness of the generator beyond the locally limited environment of the quantum device is an important feature of our concept. An external third party (e.g., dedicated public testing center) can, in principle, be equipped in large processing power (it can be a computing cloud, or even a quantum computer), to be able to publicly validate the randomness of any string – what is most important however, without disclosing the random string generated in the local generator and coupled with that verified in sharing the same level of randomness (the same entropy).

The unrestricted by resources testing of the randomness in bit sequences is crucial to verify long-ranged correlations, which commonly are unreachable by current methods strongly restricted by local computational power limitations of randomness generating setups.

In view of the mentioned problems with verification of the fidelity of randomness produced by QRNGs, we propose to utilize, in an original manner, a fundamental property of quantum systems inaccessible to classical interpretations – the quantum entanglement. We propose a simple protocol using quantum entanglement to produce a set of sequences correlated in such a way that they have the same entropy but are different (mutually independent), This allows for,

1. the transfer of the randomness testing procedure out from the QRNG setup to some trusted public third party, by publicly announcing of a single sequence, without losing, however, the secrecy of the remaining coupled sequences which share the same randomness (entropy),
2. the reduction of the average time of an arbitrary complex randomness testing (independently whether is performed locally or not) to an arbitrary smaller period of time at the cost of the increasing degree of multi-qubit entanglement.

Although idealistic model, these properties are remarkable and exceptional from a point of view of the randomness verification and overcome limits of the conventional methods, thus the development of such a new protocol or its consequences seems to be significant both of theoretical and practical reasons.

## Randomness Testing And The Shift Of The Test Procedure To An External Party

The precise definition of randomness is still elusive despite various formal mathematical approaches, which were analyzed in depth in the last century^27^. From the basic point of view, the crucial problem in a concept of randomness, in all of those approaches, is associated with an infinity limit – e.g., an infinite binary sequence length. In the case of a finite sequence there is no formal method to decide whether it is truly random or not. Let us take for example a double bit sequences 00, 01, 10 or 11 – it is not possible to decide with certainty, which of them are random and which are not. Thus when the sequence is finite, the randomness testing is limited, in fact, by checking the deviation of the frequency of occurrences of all the possible patterns with a length shorter than the sequence length, with respect to this frequency in an infinite ideal random sequence. E.g., in a truly random infinite sequence two different single-bit patterns, 0 and 1, should occur in 50% of bits, double-bit patterns, 00, 01, 10 and 11 should occur in 25% of bits, and etc.

But for an infinite sequence there exists an infinite number of possible patterns, and thus they cannot be algorithmized to form a complete (finite, i.e. computable) algorithmic randomness test. In particular, this is the main issue of the Kolmogorov (or, so called, algorithmic) randomness definition^27^.

In the case of a finite sequence the number of possible patterns scales up exponentially with the sequence length. For *n*-bit sequence the cardinality of a set of all patterns can be written as ${\sum }_{i=0}^{n-1}\,{2}^{i}$ (it is important to notice that, when verifying randomness by patterns, then the occurrences of all the patterns (that can occur more than once) must be checked; for example the sequence 01010101010101 has 50% of 0 s and 1 s, but there is an absence of double-bit patterns 00 and 11, leading to a deviation from the ideal values and randomness). This is the reason why popular randomness tests like NIST, DieHard(er) or TestU01^28–30^ utilize only a small number of mathematical tests. Those tests are specially designed to balance the execution time with the coverage of tested patterns, using various mathematical methods like e.g., Fourier transform, linear dependencies, etc. But the initial parameters suggested in these tests as default are indeed very far from covering all possible correlations which can actually occur in tested sequences (e.g., NIST Non-overlapping Template Matching Test^28^ suggests to test patterns of length up to only 10 bits – 21 bits maximally). Moreover, for example authors in^31^ state that the NIST’s state-of-the-art testing techniques are not sufficient even for pseudorandom generators. On the other hand, trying to employ a vaster range of tests batteries together with additional increase of the initial tests parameters up to theirs limits, will result in huge computing power demand (as the testing complexity scales exponentially with the complexity of correlations sought for, e.g. NIST exponential execution time increase^32^). In most cases, such requirements will not be possible to meet with the abilities of QRNG controlling unit (which commonly is a single server, PC, notebook or a smartphone), eventually resulting in an unrealistic long execution times.

We propose a new concept of shifting the randomness testing to an external party. Such party might possess enough computing power (for example be even a computing cloud) to perform randomness tests far more complex that it is possible locally within a QRNG control unit. Such external tests can detect classical correlations within the tested sequences, which would go undetectable in reasonable time by locally performed tests. As randomness tests computation scales exponentially the limit of local testing quickly expires. But the external party may not be limited by computing resources to the extent of a QRNG setup. For example, it may be a computing cloud, or can use quantumly enhanced computing units or even a quantum computer (especially in the light of the recent publication by Google team of first quantum supremacy^24^ concerning randomness testing).

But in the case of a standard quantum or classical RNG, the shifting of the randomness testing to an external party would compromise the secrecy of the generated random sequence (to test a sequence’s randomness externally one would need to publish this sequence), and make it unusable for secret e.g., cryptographic purposes. In our protocol we propose to avoid this problem, due to specific correlations (described below) between sequences generated with the use of quantum entanglement, possessing exactly the same entropy but mutually of independent binary structure. We propose to reveal only one sequence, from generated set, to a public randomness test which provides, however, the information on the randomness (entropy) of all the other sequences, which remain absolutely secret.

## The Protocol

The protocol is based on a random selection of quantum entanglement with different types of correlation. This selection is made upon the quantum measurements. As the quantum state here are defined in the computational basis $\{0,1\}$, all the considered measurements in the protocol are the measurements of an observable for which elements of the computational basis are its eigenstates, e.g., the Pauli-Z operator, ${\hat{\sigma }}_{z}$ (the eigenvalues $\{1,-\,1\}$ encoded accordingly in classical bits 0 and 1).

**Figure 1:**
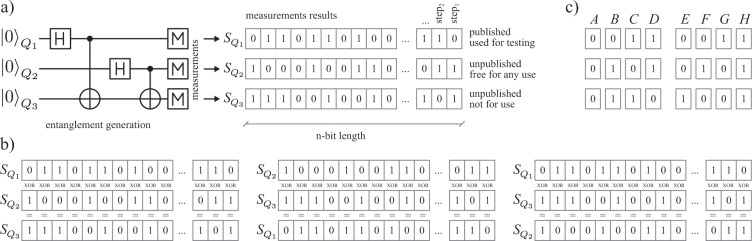
Schemes of QRNG with entanglement for three qubits protocol. (**a**) Generation, measurements and resultant sequences ${S}_{{Q}_{i}}$. (**b**) So called XOR rule – each pair of bits for any protocol step should give third bit when XOR-ed. (**c**) A,B,C,D – possible correct measurement results; E,F,G,H – incorrect results indicating bias/error of source or measurement devices.

In the simplest case of this protocol the random selection by measurement projection is performed between the correlated state $|{\Psi }^{+}\rangle =\frac{1}{\sqrt{2}}(|00\rangle +|11\rangle )$ (as the results of measurements in computational basis $\{|0\rangle ,|1\rangle \}$ are always correlated) and the anti-correlated state $|{\Psi }^{+}\rangle =\frac{1}{\sqrt{2}}(|01\rangle +|10\rangle )$ (as the results of measurements in computational basis are always anti-correlated), both from the Bell basis^12^. The initial entangled state of qubits *Q*_1_, *Q*_2_ and *Q*_3_ for this scenario, has the following form,

$$|{\psi }_{{Q}_{1}{Q}_{2}{Q}_{3}}\rangle =\frac{1}{2}(|000\rangle +|011\rangle +|101\rangle +|110\rangle ),$$

where, for example, if the qubit *Q*_1_ undergoes the measurement, then qubits *Q*_2_ and *Q*_3_ will be in correlated or anti-correlated Bell state. Subsequently, the qubits *Q*_2_ and *Q*_3_ are measured. Due to the symmetry of the initial state, in the case of ideal entanglement and perfect measurements, the order of measurements does not matter. The repetition of such a measurement procedure of 3-qubits initial state $|{\psi }_{{Q}_{1}{Q}_{2}{Q}_{3}}\rangle$ produces 3 random binary sequences, ${S}_{{Q}_{1}}$, ${S}_{{Q}_{2}}$ and ${S}_{{Q}_{3}}$ (consisting of the measurement results of qubits ${Q}_{1}$, ${Q}_{2}$, ${Q}_{3}$, respectively), cf. Fig. 1.

The classical correlation between any two of ${S}_{{Q}_{1}}$, ${S}_{{Q}_{2}}$ and ${S}_{{Q}_{3}}$ sequences is expressed by the remaining sequence. E.g., without the sequence ${S}_{{Q}_{1}}$, the sequences ${S}_{{Q}_{2}}$ and ${S}_{{Q}_{3}}$ are completely independent of each other – one (even a single bit of it) cannot be derived from the binary form of the other. This corresponds to the fact that any pair from the set $\{{S}_{{Q}_{1}},{S}_{{Q}_{2}},{S}_{{Q}_{3}}\}$, while XOR-ed (summed modulo 2), bit by bit, will result in the remaining sequence (XOR or $\oplus$ – Boolean operation called exclusive OR: $0\oplus 0=0,\,1\oplus 1=0,\,0\oplus 1=1,\,1\oplus 0=1$). This property directly follows from the form of the initial quantum state: $|{\psi }_{{Q}_{1}{Q}_{2}{Q}_{3}}\rangle =\frac{1}{2}(|000\rangle +|011\rangle +|101\rangle +|110\rangle )$, where in each ket any two bits summed module 2 will give the third bit.

On the other hand, the considered sequences are results of quantum entanglement measurements. This distinguish them from, for example, a set of three sequences, where two are produced by independent random numbers generators and the third sequence is produced by applying XOR operation on the two former ones. In such a case any two of those three sequences, while summed modulo 2, bit by bit, will give the third sequence, but e.g. the randomness of two generated independently sequences must be proven also independently. Here there was no initial quantum entanglement, which constitutes a qualitative difference from the considered set $\{{S}_{{Q}_{1}},{S}_{{Q}_{2}},{S}_{{Q}_{3}}\}$.

In the ideal case, of confirmed entanglement and perfect measurements, due to the presence of the quantum entanglement in the initial state the resultant sequences ${S}_{{Q}_{1}}$, ${S}_{{Q}_{2}}$ and ${S}_{{Q}_{3}}$ will share the same statistical properties. By using the phrase “sequences have the same statistical properties” we mean that deviations of frequencies of occurrences in sets of patterns of the same length are identical for all of those sequences in the limit of sequences length *n* tending to infinity. For finite lengths those frequencies will remain similar, i.e., if in one sequence there is 51% of 0 s occurrences and thus 49% of 1 s, by the “same statistical properties” we mean that the similar values of frequencies of occurrences will be present in other sequences, but with possible different distribution – they may concern the ratio of 0 s to 1 s or the ratio of 1 s to 0 s.

The identical statistical properties result here from quantum entanglement due to the symmetry of the initial state $|{\psi }_{{Q}_{1}{Q}_{2}{Q}_{3}}\rangle =\frac{1}{2}(|000\rangle +|011\rangle +|101\rangle +|110\rangle )$. 1) All four kets $|000\rangle$, $|011\rangle$, $|101\rangle$ and $|110\rangle$ are equally probable to be measured. 2) For each qubit ${Q}_{i}$ there are always two kets corresponding to $|0\rangle$ state of ${Q}_{i}$ and two kets corresponding to $|1\rangle$ state of ${Q}_{i}$ (e.g., for qubit ${Q}_{1}$: $|0\rangle \to |000\rangle ,|011\rangle$; $|1\rangle \to |101\rangle ,|110\rangle$). Let us call $|000\rangle \to A$, $|011\rangle \to B$, $|101\rangle \to C$, $|110\rangle \to D$. In the case of the ideal entanglement and perfect measurements, due to 1), in the infinite sequence of measured three-qubit states, the states A, B, C or D must be distributed uniformly, without any patterns (each pattern of symbols A,B,C,D of a finite length $l$ must occur in it with probability equal to 1 over the number of all the possible patterns of length $l$), and thus due to 2) the same holds to all sequences ${S}_{{Q}_{i}}$.

Thus when one of the sequences ${S}_{{Q}_{i}}$ will be proven to be random, then both remaining sequences must be considered random as well with full fidelity. In other words, due to being the result of quantum entanglement those sequences have the same statistical properties (the same entropy), though in pairs they have an independent bit structure. In contrast, this property is absent in a classical scenarios of two independently randomly generated sequences and the third sequence generated classically by applying XOR on these two former sequences (in such a situation it is not possible to prove randomness of any 2 sequences by proving the randomness of the third remaining sequence, it is only possible to prove the randomness of 1 remaining sequence by proving the randomness of the other two sequences, which, however, would not be secret anymore, as evidently derivable by applying the XOR procedure on the 2 random-proven sequences).

A crucial property of exactness of the statistical coupling of the binary sequences results from the symmetry of the involved ideal *n*-multi-qubit entanglement states (as below described upon the generalization of the proposed protocol to >3-multi-qubit case). This means that after measurements are performed on the multi-qubit entangled states, the resulting binary sequences, although are different from each other, share exactly the same statistics (entropy). This is due to the fact that the correlated or anticorrelated binary sequence positions are truly random (this fully non-deterministic randomness of correlation/anticorrelation distribution among positions in all the generated binary sequences follows directly from the symmetry of the involved multi-qubit entangled states configurations).

In considered ideal simple case of 3-qubits the entanglement decomposed symmetrically in 4 elements $\frac{1}{2}(|000\rangle +|011\rangle +|101\rangle +|111\rangle )$. The initial entanglement in such symmetric configuration yields a fully randomly either correlated or anticorrelated Bell states on the second and third qubits if the first qubit is measured. Now the series of measurements of such 3-qubit entangled states will produce a random binary sequence of measurement outcomes of the first qubits of entangled triples along with two other binary sequences of second and third qubits of the triples randomly correlated or anticorrelated as governed by the first binary sequence (in case the first qubit is measured in $|0\rangle$ then correlated and in case it is measured in $|1\rangle$ then anticorrelated). Thus those latter two binary sequences will be different but fully coupled statistically (or sharing exactly the same statistical properties, i.e., the same entropy level). The 3-qubit entangled states can be easily generalized to a multi-qubit entangled states conforming to the condition of preserving the above described symmetry.

The exact coupling or sharing the same statistical properties by all generated random binary sequences with $n$-multi-qubit entanglements is the case in a situation when $n$-multi-qubit entangled states are ideal and the measurements are perfect (no biases). In the case in which entangled states and/or measurements are not ideal, the statistical coupling will drop, but this can be countered by entanglement purification^14,16–21^ procedures for the former issue and the quantum error correction schemes^14,15^ for the latter (that in general resolve themselves to the redundancy). With entanglement purification and quantum error correction it is possible to arbitrarily closely approach the ideal case at the cost of effectiveness drop, caused by increased redundancy for the control elements of the error correction schemes.

In the imperfect situation, we can also consider some countermeasures to the following situations:

1. not properly entangled/biased initial state and ideal measurement devices,
2. perfectly entangled initial state and biased/erroneous measurement devices,
3. not properly entangled/biased initial state and biased/erroneous measurement devices.

In the case 1), due to a possible bias, the initial state could be prepared in such a manner that the resultant sequences ${S}_{{Q}_{i}}$ would not inherit identical statistical properties. For example if the initial state is prepared as $\frac{1}{\sqrt{2}}\{|000\rangle +|011\rangle \}$ then ${S}_{{Q}_{1}}$ contains only 0 s and ${S}_{{Q}_{2}}$ and ${S}_{{Q}_{3}}$ will be identical but with random distribution of 0 s and 1 s – clearly not all three sequences have the same statistical properties. To detect such imperfection one can consider random selection, in each step of the protocol, of the first qubit – that qubit which measurement result will be added to the ${S}_{{Q}_{1}}$. In this manner the bias should be uniformly distributed among 3 sequences ${S}_{{Q}_{i}}$, as statistically each of ${Q}_{i}$ will be equally often selected as the first. This would require two random bits at each step to select the first qubit among three qubits. In the case 2) when one deals with biased measurement devices the resultant sequences ${S}_{{Q}_{i}}$ may also not inherit identical statistical properties. E.g., measurement device no.1 (measuring qubit ${Q}_{1}$) may be biased to always yield 0 independently of qubit ${Q}_{1}$ real state. This will produce a ${S}_{{Q}_{1}}$ of only 0s and other sequences will definitely have different statistical properties. Thus similarly as in 1) it is important to redistribute uniformly and randomly those biases in all sequences ${S}_{{Q}_{i}}$, by randomly selecting the measurement device which will perform the last measurement of two random bits – this would be required at each step of the protocol, for this selection). This will statistically unravel those biases, due to the fact, that always the third measurement result is completely determined by the two preceding measurements results. Thus if the biased measurement device will perform the last measurement then it will statistically produce errors – the resultant 3 classical bits will violate the assumed rule (the XOR rule) that any pair of those bits while XOR-ed will always give the third one. In the case 3), the randomization of qubits numbers and measurements orders should be applied simultaneously and the results should be checked for errors violating the XOR rule, as above. As those randomizations are internal and private, thus it is possible to use for this purpose the generated in preceding generation cycle two sequences (the one published for testing, and any other unpublished, alternately concatenated). The same two random bits can be used for both selections. This requires also the initial random sequences to be used in the first protocol run – resulting not in a quantum random number generation but rather a quantum randomness expansion, allowing to statistically detect the biases or errors, either as unnatural deviation of occurrence of patterns in tested sequence, or as a violation of the XOR rule (cf. Fig. 1c)). In the case of the XOR rule violations, it also possible to verify the character of those violations, by checking (similarly as the randomness testing procedure) the occurrences of these violations along the entire sequence (with indicated bit position within this sequence where violations occurred), and specifying whether those occurrences are truly random (nondeterministic errors) or not (deterministic biases).

It is important to emphasize that if the biases/errors are distributed evenly and randomly between all the sequences, then any single sequence statistically will contain the same amount of information about the pureness of the generation process.

**Figure 2:**
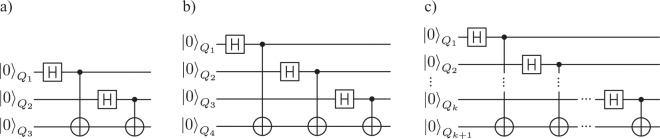
Quantum gate schemes of QRNG with entanglement for (**a**) three qubits, (**b**) four qubits, (**c**) $k+1$ qubits.

Summarizing, the protocol producing *n*-bit length sequences, in the ideal case, can be characterized in the following steps (cf. Fig. 1):

1. Preparation of the state $\frac{1}{2}(|000\rangle +|011\rangle +|101\rangle +|110\rangle )$ (e.g. cf Fig. 2a)).
2. Individual measurements of three qubits.
3. Repetition of the first two steps *n* times.
4. Obtaining three *n*-bits long sequences, ${S}_{{Q}_{1}}$, ${S}_{{Q}_{2}}$, ${S}_{{Q}_{3}}$.
5. Selecting single sequence for public announcement in order to verify it randomness by a trusted third party (as discussed above, outside and with arbitrary large computational resources) – e.g., ${S}_{{Q}_{3}}$.
6. Upon a successful randomness verification, selecting one sequence from two that are left, as a truly secret and random – e.g., ${S}_{{Q}_{2}}$.
7. The third sequence, let us call it the control sequence, here ${S}_{{Q}_{1}}$ (random as well) must, however, never be used and should be discarded (erased in an irreversible way), as its use can compromise the secrecy of the sequence ${S}_{{Q}_{2}}$. It serves as an internal proof of the fully shared statistical properties (and the XOR rule) between the sequences ${S}_{{Q}_{2}}$ and ${S}_{{Q}_{3}}$ and holds the full information about deriving sequence ${S}_{{Q}_{2}}$ from ${S}_{{Q}_{3}}$ or vice versa.

The intact secrecy of the unpublished sequences (which were proven to be random) is the factual situation, in which the knowledge of the whole published sequence does not reveal any bit of the unpublished sequences. This results from the construction of the sequences ${S}_{{Q}_{i}}$ as for every of theirs *p*-th elements one can write the following (let us call this the XOR rule),

$${S}_{{Q}_{i}}^{(p)}\oplus {S}_{{Q}_{j}}^{(p)}={S}_{{Q}_{k}}^{(p)},\,{\rm{for}}\,i,j,k=1,2,3,i\ne j,i\ne k,j\ne k.$$

The knowledge of the resulting bit from the XOR operation, does not give any information on the exact value of bits, which were XOR-ed. It informs only whether they were the same or not, thus without additional knowledge on any of unpublished bits it is useless. This is the reason why, in the proposed protocol, one of two unpublished sequences must not be used publicly, and should be discarded – to disallow any potential eavesdropper to gain additional knowledge.

The XOR reduction is commonly acknowledged as the prototype of privacy amplification procedures for the key distillation in the quantum key distribution/expansion protocols. When the eavesdropper knows a single bit value from the set of two bits ${b}_{1},{b}_{2}$ (irrespectively, whether it is ${b}_{1}$ or ${b}_{2}$), then by XOR-ing those two bits, $a={b}_{1}\oplus {b}_{2}$, one removes any knowledge of the eavesdropper about the resultant bit $a$. With the knowledge of only a single ${b}_{i}$ the value of the bit $a$ is fully dependent on the other bit $b$, which was unknown to the eavesdropper.

## The Protocol Generalization

It is possible to straightforwardly extend presented protocol to generate any number of classically independent sequences (by providing a corresponding number of entangled qubits). Then the randomness of all those sequences can be can be proven by analyzing only one of them. This leads to a possibility of lowering an average execution time of any complex randomness testing to an arbitrary small period of time (testing time of a single sequence will remain the same but the number of sequences, which will be instantly verified in a security non-compromising manner by the test procedure and its result, can by arbitrarily large). This is a very profound property of the protocol following from the adequately configured quantum entanglement utilization.

To extend the considered protocol by one sequence, one additional qubit must be entangled with the previous state, as presented on the scheme depicted in Fig. 2. The new qubit undergoes Hadamard operation and then controls within the controlled-NOT operation the last qubit from the previous state (cf. Fig. 2c)). Thus four-qubit state (cf. Fig. 2b)) will have the following form $|{\psi }_{{Q}_{1}{Q}_{2}{Q}_{3}{Q}_{4}}\rangle$ = $\frac{1}{2\sqrt{2}}[|0\rangle (|000\rangle +|011\rangle +|101\rangle +|110\rangle )$ + $|1\rangle (|111\rangle +|100\rangle +|010\rangle +|001\rangle )]$ = $\frac{1}{2\sqrt{2}}[(|00\rangle +|11\rangle )(|00\rangle +|11\rangle )$ + $(|01\rangle +|10\rangle )(|01\rangle +|10\rangle )]$.

The symmetry of this state is similar to that of the three-qubit state. If ${S}_{{Q}_{1}}$, ${S}_{{Q}_{2}}$, ${S}_{{Q}_{3}}$ and ${S}_{{Q}_{4}}$ denotes the $n$-bits sequences being the results of four qubits measurements repeated $n$ times, then the sum modulo 2 (XOR operation) bit by bit of any three of them will give the fourth sequence, ${s}_{{Q}_{2}}^{(i)}+{s}_{{Q}_{3}}^{(i)}+{s}_{{Q}_{4}}^{(i)}\,{\rm{mod}}\,2={s}_{{Q}_{1}}^{(i)}$, where ${s}_{{Q}_{j}}^{(i)}$ denotes the $i$-th bit of $j$-th sequence. Thus removing a single sequence from this set of four makes it impossible to recover those correlations, what guarantees the mutual secrecy of three sequences that are left (even when adversary will get to know any two of them the third one will still remain secret). But more importantly, all of those sequences share the same statistical properties – this allows to verify (publicly or locally) the randomness of only a single sequence and the result of this verification will concern the others sequences as well. In a case of successful verification of randomness of a single sequence all other sequences are considered to be random.

From the point of view of randomness testing the four-qubit entanglement protocol scenario (with three random sequences) shortened the average time of testing by half in comparison to the three-qubit entanglement protocol scenario. It is possible to scale up the generating setup in terms of multi-qubit entanglement. To obtain an arbitrary number $k$ ($k\ge 2$) of sequences that are classically completely independent from each other, but with identical statistical properties, it is needed to prepare a quantum state consisting of $k+1$ appropriately entangled qubits (as presented further). Thus the testing time will be divided by $k-1$ in case of public announcement for testing of a single sequence. In this manner one can make an average time of randomness testing (i.e., the total time of testing of a single sequence divided by the number of secret sequences assessed by this testing result) for all generated sequences shorten as 1/*k* with increasing $k$ (in fact $k+1$, which is the number of needed qubits that are appropriately entangled).

Finally, the initial quantum state for generation of $k$ sequences, consisting of $k+1$ entangled qubits, has the following form $|{\psi }_{{Q}_{1}\ldots {Q}_{k+1}}\rangle ={2}^{-\frac{k}{2}}(\mathop{\prod }\limits_{i=1}^{k}\,\mathop{\sum }\limits_{{q}_{i}=0}^{1}\,|{q}_{i}\rangle )\,|{q}_{1}\oplus {q}_{2}\oplus \cdots \oplus {q}_{k}\rangle$, where $\oplus$ is the sum modulo 2. After selection of one qubit as a control qubit (and discarding its sequence of measurement results) the series of measurement results of other $k$ qubits will be not correlated (which means that it is impossible to obtain one from the other without knowing the relevant control sequence storing the full information about this correlation), but will have the same statistical properties (the same entropy).

Similarly as in the 3-qubit case, in the $k$-qubit scenario a bias/error detection mechanism, by mixing the control qubit choice and the last measurement choice, can be considered. Here, as the number of qubits is higher, then accordingly the higher the number of random bits, required to those random selections, will be. As the number of required random bits, in a single step for $k$-qubit scenario, should allow to enumerate each number up to $k$ (to select any from $k$ qubits and $k$ measuring devices), thus it scales as ${\log }_{2}\,{k}_{2}$, where ${k}_{2}$ is the power of 2 closest to $k$ (but greater or equal). It will reduce the number of generated publicable sequences, to $k-lo{g}_{2}{k}_{2}$, as the unpublishable sequences, like control sequence, can also be used for those private random selections, as discussed in the 3-qubit case.

## Summary

An infringement of ideally nondeterministic character of quantum random number generator process by inevitable imperfections of physical implementation, may pave a trace for the determinism, in the form of long-range classical correlations, which are very difficult to be detected by conventionally used randomness tests (e.g., problems with NIST test suites^31,32^, or even known to be deterministic PRNGs successfully passing the TestU01 suit^33^).

We proposed a new protocol which takes an advantage, as above demonstrated by its generalization to the $n$-qubit entanglement configuration, from the ability to exponentially reduce the local computational resources by the splitting of the final binary random sequence into arbitrarily many sequence parts of equal length and sharing exactly the same statistical properties due to the quantum arbitrariness of the correlated/anticorrelated entangled states involved. All are different but coupled in a way, that they all have the same entropy. This means that the checking of the entropy on only one such string, confirms the entropy of all the remaining. Additionally all these sequences are mutually independent (underivable from each other, without the knowledge of control sequence) due to symmetries of the involved multi-qubit entanglements. These properties allow to arbitrarily down-scale computational resources needed to verify randomness (or measure of the entropy) at the cost of the growing number of qubits in a multi-qubit entangled state. We have thus separated the entropy measure from exponential computational difficulty of finding repeating patterns, employing the specific multi-qubit entanglement properties, which eventually lead to an arbitrarily high reduction of the exponentially-hard binary sequence randomness verification problem. Additionally, the disclosure of one of the sequences does not disclose or reduce the secrecy of other ones (coupled sequences). This allows for a safe verification of the randomness (or the entropy measurement) of both (or more) sequences in the pair (set) by testing only one coupled to them. We thus separate the entropy measure form the secrecy.

Thus the proposed protocol of public testing of the randomness of the ‘ideal-quantum random copy’, without disclosing the privacy of the original sequence is an important advance, especially in view of recent rapid development in the engineering of multi-qubit entanglement device implementations and control as demonstrated e.g. by the Sycamore quantum processor achieving quantum supremacy as claimed by Google (2019)^24^. It should be emphasized that the similar as described above trading of the increase of the $n$-multi-qubit entanglement level for reducing the problem of randomness verification of the binary sequence has played a central role in the quantum supremacy result. The success in implementation of the Sycamore processor supports thus the feasibility of the presented our proposal. It should be noted that we have announced the main idea how to employ $n$-qubit entanglement as in our protocol in 2017^25^, earlier than the Sycamore architecture has been revealed.

Nevertheless, Google’s Sycamore implementation could be also the external public party, which could perform testing of the randomness of the disclosed ‘ideal random copy’ of the bit string generated by the QRNG arranged according to our protocol (in a not-generalized option), which additionally supports the feasibility of its practical effective realization. The generalized $n$-multi-qubit entanglement protocol would not need Google’s Sycamore processor as it would implement fully by itself the arbitrary high reduction of exponential computational scaling of entropy measurement.

Applying the proposed protocol, even in its simplest option, it is always possible to shift the randomness testing to an external party (or to multiple independent parties simultaneously, to minimize the risk of a collusion) by publicly announcing one of the sequences for an external and open randomness verification, without losing the secrecy of the other generated sequences. Furthermore, the possibility of shortening of an average time needed for testing execution (locally or not) up to an arbitrary small value in theory (with zero limit, when the number of correlated sequences tends to infinity), by testing only one from a whole family of correlated sequences, seems to be an interesting and important feature by itself. Observing the recent substantial development of experiments with controlling of entangled complex quantum system (consisting of quantumly entangled 18 or 20 qubits^34,35^, or more recently 53 qubits^24^), one may reasonably expect that many-qubits entanglement based protocols, like the one described, would be feasible to implement in the future, despite all technical difficulties^34,35^ on the way to scale up a controllable and fault-tolerant multi-qubit entangled states.
