# Author Correction: Quantum random number generators with entanglement for public randomness testing

**DOI:** 10.1038/s41598-020-65142-6

**Published:** 2020-05-12

**Authors:** Janusz E. Jacak, Witold A. Jacak, Wojciech A. Donderowicz, Lucjan Jacak

**Affiliations:** 1 Department of Quantum Technologies, Faculty of Fundamental Problems of Technology, Wrocław University of Science and Technology, Wyb. Wyspiańskiego 27, 50-370 Wrocław, Poland; 2 CompSecur, Pilsudskiego 74/309b, 50-020 Wroclaw, Poland

Correction to: *Scientific Reports* 10.1038/s41598-019-56706-2, published online 13 January 2020

This Article contains typographical errors in the Acknowledgment section.

“Supported by the NCBiR project PP3/W-32/D-2223/2014 Jurand.”

should read:

“Supported by the NCBiR project POIR.01.01.01-00-0173/15 Jurand.”

